# Biochar Amendment Increases Peanut Production Through Improvement of the Extracellular Enzyme Activities and Microbial Community Composition in Replanted Field

**DOI:** 10.3390/plants14060922

**Published:** 2025-03-15

**Authors:** Cheng Liu, Shijie Shang, Chao Wang, Jing Tian, Liting Zhang, Xiaoyu Liu, Rongjun Bian, Qunling He, Fengye Zhang, Lei Chen, Marios Drosos, Muhammad Azeem, Lianqing Li, Shengdao Shan, Genxing Pan

**Affiliations:** 1 Key Laboratory of Recycling and Eco-Treatment of Waste Biomass of Zhejiang Province, School of Environment and Natural Resources, Zhejiang University of Science and Technology, Hangzhou 310023, China; chengliu01@zust.edu.cn (C.L.);; 2 Institute of Resource, Ecosystem and Environment of Agriculture and Department of Soil Science, Nanjing Agricultural University, Nanjing 210095, China; 3 Institute of Cash Crop, Shangqiu Academy of Agriculture and Forest Sciences, Shangqiu 476002, China; 4 Department of Agricultural, Forest, Food and Environmental Sciences, University of Basilicata, Viale dell Ateneo Lucano n. 10, 85100 Potenza, Italy; 5 State Key Laboratory for Ecological Security of Regions and Cities, Ningbo Observation and Research Station, Institute of Urban Environment, Chinese Academy of Sciences, Xiamen 361021, China

**Keywords:** peanut, yield and quality, monoculture, biochar, enzyme activity, microbial community

## Abstract

Peanut yield and quality are often threatened by soil degradation under continuous cropping. Biochar has been known to improve the soil microbial community and plant resistance. However, studies on its functions to reduce soil degradation losses and improve the peanut yield are limited. A field peanut experiment was conducted in an Alfisol soil and biochar was applied at a rate of 20 t ha^−1^ in 2022. The biochar was prepared from woodchip (WB) and maize straw (MB) feedstocks alone, as well as with co-composted biochar of the same feedstocks with pig manure labeled as WBSC and MBSC amendment, respectively. The conventional organic manure was applied as a control treatment (OM). All plots were base-fertilized with a mineral compound fertilizer of N-P_2_O_5_-K_2_O (16-16-16, %) at 600 kg ha^−1^. Topsoil (20 cm) and plant samples were collected at the time of peanut harvest. Soil quality, enzyme function, peanut growth traits, microbial abundance, and community composition were analyzed. Compared to OM, peanut yields increased by 22%, 23%, and 18% under WB, WBSC, and MBSC, respectively. The content of oleic acid increased by 4–5%, while the content of linoleic acid decreased by 7–9%, respectively, under biochar–compost treatments. However, biochar amendment alone showed non-significant changes in these fatty acids. The soil extracellular enzyme activity increased by 3.7–5.5% with biochar amendments and 6.4–10.1% with biochar–compost application. The enzyme activity ratio of hydrolase to non-hydrolase, of C cycling to N cycling, and of P cycling increased by 11.4–15.9%, 20.9–33.8%, and 14.7–23.5% under biochar amendments and by 20.5–25.0%, 17.4–39.0%, and 23.5–32.3% under biochar–compost, respectively. Overall, crop residue biochar enhanced peanut yield and quality by improving soil aggregation, enzyme functionality, and fungal community in line with the soil nutrient supply.

## 1. Introduction

Soil health is crucial as it supports a vital living ecosystem that sustains plant growth and ensures a reliable food supply [1]. However, global soils have been at risk due to extensive soil degradation, along with climate change, biodiversity loss, and environmental pollution [2]. Currently, soil-borne diseases have become a serious problem in the production of continuously replanted annual crops, which often results in growth obstacles for the plants to harvest [3]. Even though the replanting problem is typical, adverse plant–soil feedback results in crop yield and grain quality reduction [4]. Peanuts are increasingly grown on the same land without crop rotation due to limited amounts of arable land and intensive regional agro-industrialization. Such intensive peanut production is particularly widespread in the subtropical regions of China. However, its consecutive monoculture has caused a continuous decrease in yield and quality and increased susceptibility to diseases [5,6,7].

To overcome soil-borne diseases, farmers often utilize fungicides and/or organic fertilizers in continuous cropping fields. The application of fertilizer, manure, or compost to soils often leads to the rapid loss of organic carbon and available N and P, incurring financial costs for the farmer and causing the leaching of essential plant nutrients [8]. This process can result in environmental pollution. In the last decade, it has been widely reported that biochar soil amendment plays diverse roles in crop productivity enhancement, carbon sequestration, greenhouse gas emission mitigation, soil fertility improvement, and contamination control [9]. Even though the benefits of biochar applications have been widely reported, the economic feasibility of high biochar due to cost and the difficulties during application restrict large-scale implementation of biochar worldwide.

Recently, it has been noticed that biochar has been proposed for use as a novel composting additive to enhance the composting process [10]. Biochar can extend the residence time of compost in the soil and enhance nutrient retention due to its small particle size and high adsorption capacity. However, biochar itself lacks readily available nutrients for plants, while compost supplementation ensures a sufficient nutrient supply [11]. Co-composting with biochar ultimately contributes to compost quality, including nutritional value, safety, and stability [12]. The co-composting process modifies biochar surface functional groups, nutritional content, cation exchange capacity (CEC), and the amount of inhibitory aromatic compounds [13]. Therefore, combining biochar with compost may offer a potential solution to overcome these issues. Previous findings revealed that co-composted addition to soil can help to improve soil health and increase plant growth, as tested for other crops. For example, Kammann et al. [14] reported that the biomass yield of poor soils was dramatically raised by 305% after applying co-composted biochar (2%, *w*/*w*), but the biomass yield was decreased with biochar addition alone. Jien et al. [11] suggested that compost and biochar were more effective in stabilizing and sequestering carbon than biochar alone. Meanwhile, the interaction between biochar and compost in combined applications has received growing attention [13,15]. These changes can potentially increase the efficiency of biochar and compost in soil improvement. Given these, biochar–compost has been considered as a potentially more effective soil amendment than application of biochar or compost alone. However, despite a rudimentary understanding of some of the advantages of applying biochar–compost, it is still unclear how biochar–compost treatments address different soil issues. The practical application of biochar–compost is severely hampered by this, and farmers would prefer to stick with the previous simple biochar or compost amendments alone rather than by their combined application [13].

To date, co-composted biochar has not been tested on peanut crops to address replanting issues and soilborne diseases. This innovative approach could improve soil health by enhancing nutrient retention, increasing microbial diversity, and suppressing harmful pathogens. By integrating biochar with compost, it is expected to not only mitigate replanting challenges but also promote healthier soil ecosystems, ultimately improving peanut yield, grain quality, and resistance to soilborne diseases. We hypothesize that (i) biochar and biochar–compost may synergically increase the yield and quality of peanuts by providing soil nutrients, which will improve soil structure, microbial community and functions; and (ii) biochar–compost may be a potentially more effective soil amendment for replanted fields than biochar alone. To test the effect of biochar and co-compost on the quality and yield of peanuts, a short-term field experiment was established in May 2022. This study aimed to provide a new insight into utilizing biochar–compost for soil improvement compared to biochar or compost alone.

## 2. Results

### 2.1. Peanut Yield and Kernel Quality

Data on peanut yield and yield components are organized in Table 1. Compared with OM, peanut yields increased (*p* < 0.05) by 22%, 23%, and 18% under WB, WBSC, and MBSC treatments, respectively, and by 14% (*p* > 0.05) under MB treatments. Application of biochar and biochar–compost increased peanut plant height on average significantly but slightly (by 6–9%), while the pod number per plant was unchanged under biochar treatment but increased by 45% and 35% under WBSC and MBSC treatment, respectively. The pod weight per plant increased by 39% and 33% under WBSC and MBSC treatments, respectively. There were no significant differences in peanut kernel weight among the different treatments but the kernel weight was significantly increased by 15% under WB treatment. However, the kernel-to-pod ratio was significantly increased under biochar and biochar–compost, with an average increase of 4–10%. In particular, the peanut survival rate was increased by 6.5–7.4% under biochar–compost treatment, though it was unchanged under other treatments.

Table 2 shows changes in peanut kernel quality. There was no significant change in crude fat among the different treatments, but the content of protein was 11% higher with MB than with OM treatment. Compared to OM, the content of oleic acid was significantly but slightly (by ca 5%) increased. Contrarily, linoleic acid content decreased by 7–9% under the biochar–compost treatments, but there was no significant change under the biochar amendments. The contents of total sugar and sucrose were insignificantly changed among treatments. In contrast, the contents of soluble sugar were increased by 22%, 43%, and 13% under WB, MB, and MBSC, respectively, but insignificantly changed under WBSC, respectively, over OM treatment.

### 2.2. Soil Properties

The properties of topsoil are described in Table 3. Soil pH was significantly elevated by 0.88 and 0.85 units, whereas soil bulk density was unchanged under all the biochar-based treatments compared to OM. The content of SOC was increased by 67% and 68% under WB and MB, respectively, while it remained unchanged under biochar–compost treatments compared to OM. Differently, the content of total N was significantly increased by ca 20% under WBSC and MBSC, while it insignificantly changed under WB and MB. In addition, available P increased by 20.2% under MB while it remained unchanged with WB. Moreover, biochar amendment increased available K by 21.3–37.3% over OM. Soil CEC was unchanged with all the biochar-based treatments. The content of MBC increased by 18.5%, 29.3%, and 37.4% under MB, WBSC, and MBSC but remained unchanged under WB, respectively, compared to OM. MBN significantly increased by 28.7% under WBSC.

**Figure 1:**
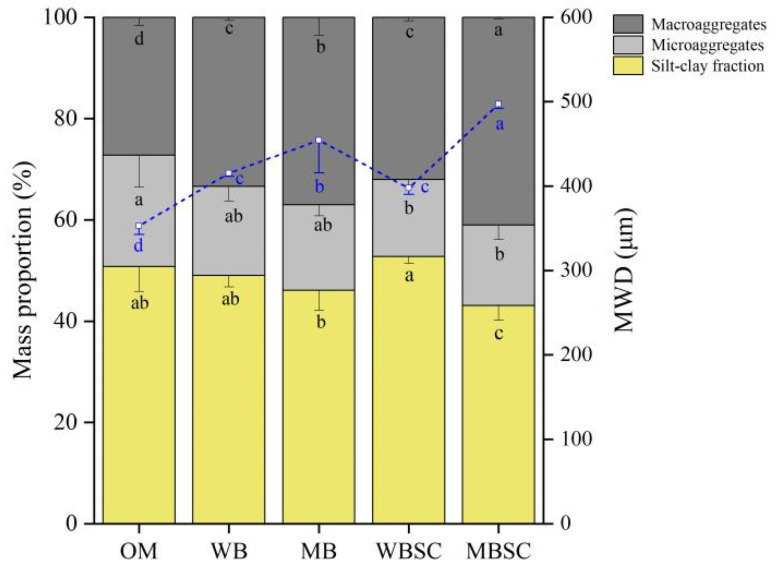
Mass proportion of size fractions of water stable aggregates and calculated mean weight diameter (MWD) of topsoil following amendment under different treatments. Different letters under the bars indicate significant differences (*p* < 0.05) among the treatments. The error bars represent standard deviations (n = 3). OM, amendment at 20 t ha^−1^ of conventional organic manure; WB and MB, amendment at 20 t ha^−1^ of woodchip biochar and maize straw biochar respectively; WBSC and MBSC, amendment at 20 t ha^−1^ of co-compost of woodchip biochar and pig manure, and co-compost of maize straw biochar and pig manure respectively.

Data of the mass proportion of size fractions of water stable aggregates and the calculated mean weight diameter (MWD) are presented in Figure 1. The silt–clay fraction was decreased by 15.2% under MBSC, while no changes were observed under other treatments compared to OM. The microaggregates decreased by 31.3% and 28.4% under WBSC and MBSC, while the macroaggregates increased by 23.7%, 35.8%, 17.8%, and 50.6% under WB, MB, WBSC, and MBSC, respectively. The MWD increased by 28.7%, 13.2%, and 40.1% under WB, MB, WBSC, and MBSC versus OM, respectively.

### 2.3. Soil Extracellular Enzyme Activities and Function

Changes in the soil extracellular enzyme activities are shown in Table 4. The activities of α-Glucosidase were increased by 18.9–43.8% with biochar amendment and by 43.8–115.8% with biochar–compost amendment. The activities of β-Glucosidase were increased by 37.4–45.6% under biochar amendment and by 45.5–60.1% under biochar–compost amendment. Similarly, the activities of β-Xylosidase were increased by 44.8–45.8% under biochar amendments, and by 80.3–87.7% under biochar–compost amendments. The activities of β-Cellobiohydrolase increased by 41.9% and 41.2% under WB and MBSC, respectively, but no significant changes were spotted under MB and WBSC. The activities of N-acetyl-glucosaminidase and acid phosphatase increased by 7.1–10.1% and 14.4–15.5% under biochar amendment, and by 18.0–20.1% and 14.8–23.3% under biochar–compost amendments, while the activities of sulfatase insignificantly changed under all treatments. For polyphenol oxidase and peroxidase, the activities of polyphenol oxidase were unchanged among all treatments, while the activities of peroxidase increased by 27.5–33.9% under biochar treatments and by 25.1–66.1% under biochar–compost treatments, respectively. In addition, the diversity of the enzyme activity increased by 3.7–5.5% under biochar amendments and by 6.4–10.1% under biochar–compost. The hydrolase/non-hydrolase, C/N cycling, and C/P cycling increased by 11.4–15.9%, 20.9–33.8%, and 14.7–23.5% under biochar amendments and 20.5–25.0%, 17.4–39.0%, and 23.5–32.3% under biochar–compost treatments.

### 2.4. Gene Abundance and Community Composition of Rhizosphere Microbiome

**Figure 2:**
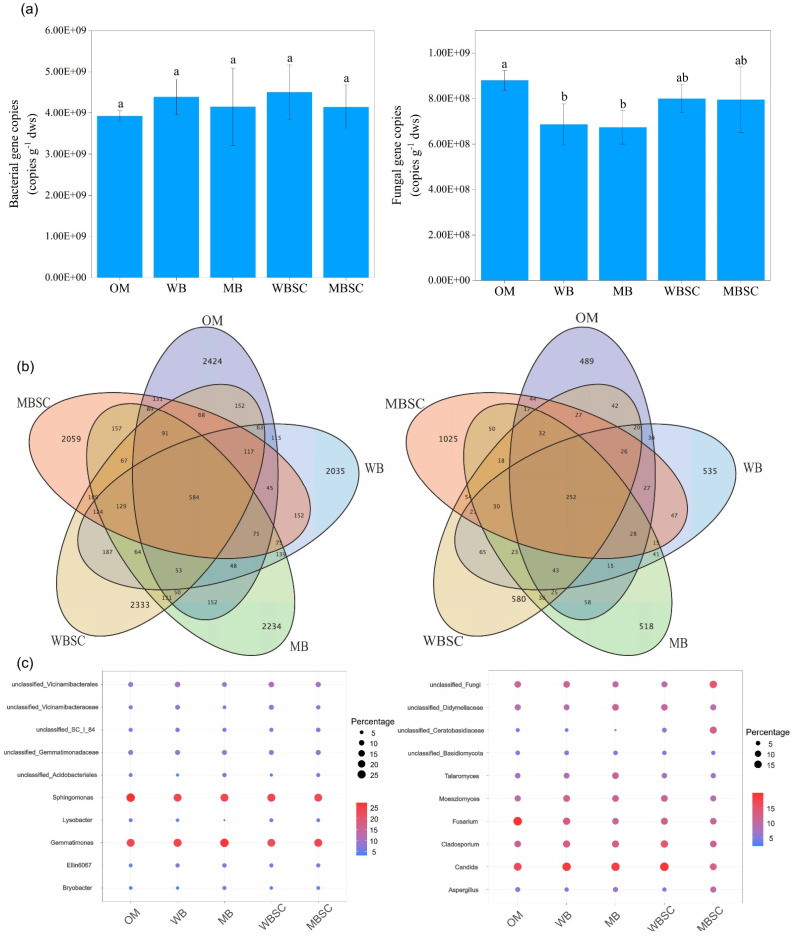
Gene abundance (**a**), exclusive and shared OTUs at the genus level (**b**), and top 10 phyla composition (**c**) of bacteria (**left**) and fungi (**right**) in peanut soil, sampled at harvest, following soil amendment. Different letters over the bars indicate significant differences (*p* < 0.05) among the treatments. The error bars represent standard deviations (n = 3). OM, amendment at 20 t ha^−1^ of conventional organic manure; WB and MB, amendment at 20 t ha^−1^ of woodchip biochar and maize straw biochar respectively; WBSC and MBSC, amendment at 20 t ha^−1^ of co-compost of woodchip biochar and pig manure, and co-compost of maize straw biochar and pig manure respectively.

Data of the total gene abundance and the community composition of bacteria and fungi of rhizosphere soil are organized in Figure 2. The bacterial gene copies showed no significant difference among the treatments, while the fungal gene copy number was significantly decreased by 22.0% and 23.5% under WB and MB compared with OM, respectively. The shared OTUs number of bacteria among all treatments was 584, and the exclusive OTU numbers under OM, WB, MB, WBSC, and MBSC were 2424, 2035, 2234, 2333, and 2059, respectively. The shared OTUs number of fungi among all treatments was 252, and the exclusive OTU numbers under OM, WB, MB, WBSC, and MBSC treatments were 489, 535, 518, 580, and 1025, respectively. As Figure 2c shows, the top ten genera for fungal abundance are Candida, Cladosporium, Fusarium, unclassified_Fungi, Moesziomyces, unclassified_Didymellaceae, Aspergillus, unclassified_Ceratobasidiaceae, unclassified_Basidiomycota, and Talaromyces, respectively. The top 10 genera are Sphingomonas, Gemmatimonas, unclassified_Vicinamibacterales, unclassified_Gemmatimonadaceae, unclassified_Vicinamibacteraceae, unclassified_SC_I_84, Ellin6067, unclassified_Acidobacteriales, Bryobacter, and Lysobacter.

### 2.5. Diversity and Community Composition of Rhizosphere Microbiome

**Figure 3:**
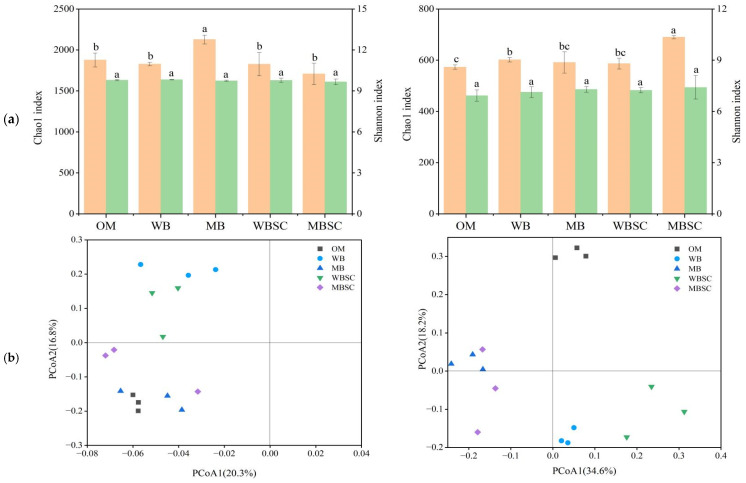
Microbial diversity (**a**) and principal coordinate analysis (PCoA) ordinations of bacteria (**left**) and fungi (**right**) community composition (**b**) based on unweighted UniFrac distance metric in peanut soil, sampled at harvest, following soil amendment. Different letters over the bars indicate significant differences (*p* < 0.05) among the treatments. The error bars represent standard deviations (n = 3). OM, amendment at 20 t ha^−1^ of conventional organic manure; WB and MB, amendment at 20 t ha^−1^ of woodchip biochar and maize straw biochar respectively; WBSC and MBSC, amendment at 20 t ha^−1^ of co-compost of woodchip biochar and pig manure, and co-compost of maize straw biochar and pig manure respectively.

As Figure 3 shows, the bacterial Chao1 index increased significantly under MB treatment compared to OM treatment, but the change under other treatments was not significant. The fungal Chao1 index increased significantly under WB and MBSC treatments, while the Shannon index of bacteria and fungi did not change significantly between treatments. The results of principal coordinate analysis based on the unweighted UniFrac distance measure showed that the bacterial community structures were not significantly differentiated among treatments, while the fungal community was completely separated from MB and MBSC at PCoA1, and OM was completely separated from WB and WBSC at PCoA1.

**Figure 4:**
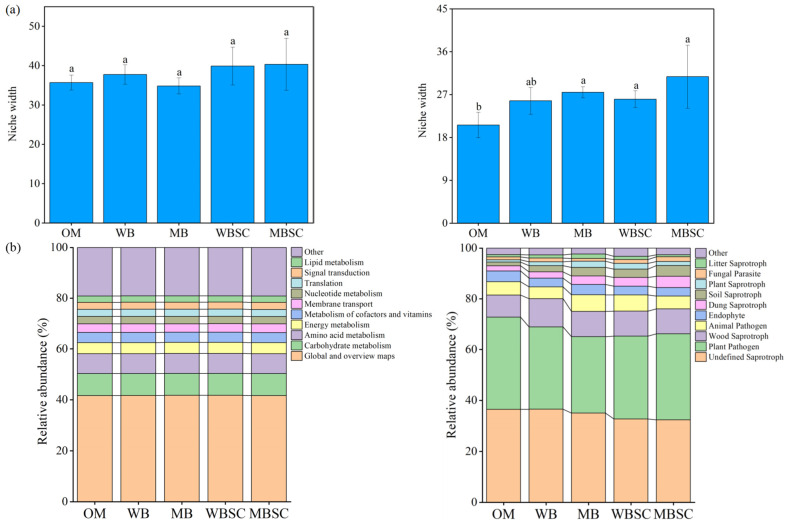
Mean habitat niche breadth (**a**) and functional traits (**b**) of bacterial (**left**) and fungal (**right**) community in peanut soil, sampled at harvest, following soil amendment. Functional traits of the bacterial community on KEGG pathway (KO tier 2) and functional traits of fungal community using FUNGuild with OTU data in peanut soil, sampled at harvest, following soil amendment. Different letters over the bars indicate significant differences (*p* < 0.05) among the treatments. The error bars represent standard deviations (n = 3). OM, amendment at 20 t ha^−1^ of conventional organic manure; WB and MB, amendment at 20 t ha^−1^ of woodchip biochar and maize straw biochar respectively; WBSC and MBSC, amendment at 20 t ha^−1^ of co-compost of woodchip biochar and pig manure, and co-compost of maize straw biochar and pig manure respectively.

The average niche widths of bacteria and fungi were calculated according to the Levis niche width theory, as Figure 4 shows. The bacterial niche did not change significantly among treatments, while the fungal niche widths increased by 33%, 26%, and 49% under MB, WBSC, and MBSC treatments, respectively, compared to OM treatment, but did not change significantly under WB treatment. As Figure 4b shows, KEGG pathway analysis of bacterial communities showed no difference in secondary classification (KO tier 2) among treatments. The FunGuild-based fungal community function study found that the relative abundance of biochar and biochar–compost plant pathogens decreased, while the relative abundance of soil saprotroph and plant saprotroph increased.

## 3. Discussion

Long-term continuous monoculture can lead to a severe decrease in peanut yield, as shown in previous studies [5,16]. In this study, overall, biochar and biochar–compost could increase peanut survival rate, peanut yield, and kernel quality, increase C, N, and P in soils, and stabilize soil aggregates, as well as improve fungal diversity and bacterial and fungal community. Among the above-mentioned indicators, biochar–compost amendment is more effective than biochar or manure compost alone. This is consistent with our hypothesis. The potential of applying biochar or compost alone for peanut soil improvement has been demonstrated in many studies [17,18,19], but the combined application of the two materials created even greater value for replanted peanut soil improvement in this study. Co-composting biochar with organic waste benefits the compost, the composting process, and the biochar itself by increasing its aging processes [10]. Biochar is widely regarded as an effective strategy for soil carbon sequestration, and biochar–compost might be a more promising and economically beneficial amendment than biochar alone. The high cost of biochar limits its use in soil on a large scale [20]. The combination of biochar and cheap manure compost not only lowers the input of biochar and thus reduces the material cost, but also greatly reduces biochar losses and disposal costs during on-site applications [21].

### 3.1. Biochar–Compost Synergistically Improves Peanut Production and Soil Quality

A meta-analysis revealed cereal crops (barley, maize, oat, quinoa, and wheat) showed a significant increase in grain yield of 39.7% under biochar–compost application compared to unamended biochar–compost treatments [10]. While fertile soils may not benefit from the biochar–compost application, a negative response could be due to the dilution effect of the amendment on soil nutrients [10,22]. In this study, biochar–compost increased peanut pod yield by 18–23% (Table 1). On poor soil with an SOC of 8.60 g kg^−1^ and a total N of 0.35 g kg^−1^ where peanuts are continuously planted, the application of biochar–compost shows great advantages in increasing the peanut yield. Moreover, the phenotypic growth of peanuts was also significantly enhanced, in which plant height, pod number, and pod weight per plant were increased to varying degrees. The application of biochar–compost serves as a partial substitute for chemical fertilizers due to the direct supply of nutrients and the enhancement of nutrients’ cycling and availability [12]. In this study, under the biochar–compost treatment, soil total N was significantly increased by 20–22%, MBC was increased by 29–37%, and MBN was increased by 17–29%. Similarly, the addition of 40 Mg ha^−1^ of biochar–compost to a low fertility tropical Ferralsol increased maize grain and biomass production by 10–29% and 9–18%, respectively, when compared to inorganic fertilizers [23,24].

Biochar–compost usually shows higher total nitrogen (N) than it does without biochar. However, as a second limiting nutrient in soil following N, the phosphate content in biochar compost varies among studies [25,26]. The improvement of phosphate content in compost with biochar was mostly due to high phosphorus reserves (P) in the added biochar. Some nutrients, such as K^+^, Ca^2+^, and Mg^2+^, showed improved retention with biochar amendment due to their adsorption by the negatively charged biochar surface. Although in this study, compared with traditional compost, biochar-based organic fertilizer did not significantly increase the content of available phosphorus and potassium, it did significantly increase the content of trace elements, such as Fe, Zn, and Mo (Table S2). The peanut growth process requires the absorption of various elements, especially Fe and Mo. As legumes, peanuts can fix dinitrogen (N_2_) by establishing mutualistic symbiosis with compatible rhizobia [27]. Molybdenum-iron protein is the active site of nitrogenase [28]. Previous studies have demonstrated that Fe deficiency causes serious chlorosis, inhibiting the growth of peanut seedlings and decreasing the concentration of soluble Fe and chlorophyll in peanuts simultaneously [29]. Liu et al. [18] found that applying maize straw biochar and rice husk biochar at 20 t ha^−1^ increased the content of available Fe and Mo in peanut soil, promoted the N fixation of peanuts, and thus increased the peanut yield, as noticed in this study. In addition, biochar–compost not only improves the peanut yield and soil quality but also the peanut quality. Compared to traditional compost, biochar and biochar–compost notably improved peanut quality, with the significant alteration being a 4–5% increase in oleic acid content, a 7–9% decrease in linoleic acid, and the contents of soluble sugar increased by 13–43%. Qu et al. [30] confirmed that supplementing peanut soil with Fe or Mo can enhance oleic acid and soluble sugar levels by regulating the metabolic pathways of peanuts. In this study, the increase of Fe and Mo in the soil after applying biochar–compost may be one of the reasons for the improved peanut quality. Yuan et al. [31] also found individual amino acids significantly increased by 10.2% on average after maize straw biochar (450 °C) application at a rate of 1% (*w*/*w*) into the coastal soil. However, when 3 kg biochar–compost with 15% or 30% forest wood residue biochar was applied into the planting holes prior to replanting apple trees, both the growth and quality of apples were not enhanced [32]. It may be that biochar–compost is less effective for perennial crops than annual peanuts, or that soil fertility is not a limiting factor in European apple orchards. The mechanism of how biochar–compost improves crop quality needs a lot of further exploration.

### 3.2. Microbial Manipulation and Enzyme Activity Shifted with Biochar–Compost

The decline in the SOC, soil structure, and nutrients caused by continuous monocropping have substantial impacts on the microbial community composition, which controls enzymatic activity [33,34]. These environmental factors reduced the relative abundance of beneficial bacteria (i.e., *Bacillus*) capable of releasing polyketides and antibiotics to inhibit pathogenic microbes [35,36], thereby allowing pathogenic fungi to accumulate. However, in this study, although the application of biochar and biochar–compost had no significant effect on the total copy number of bacteria, and the copy number of fungi decreased under biochar treatment, the community of bacteria and fungi changed significantly (Figure 2). When conventional compost, biochar, and biochar–compost were each applied, the bacterial community showed no significant changes. In contrast, the fungal community was completely distinguished between OM and other treatments (Figure 3b). Not only that, but the diversity of fungi was also significantly increased under the treatment of biochar and biochar–compost. In particular, among the top ten abundance of fungi, the abundance of pathogenic *Fusarium* decreased significantly. *Fusarium* is one of the primary pathogens of continuous cropping obstruction of peanuts, resulting in a severe reduction in peanut yield [5]. This echoes our findings that biochar and biochar–compost improve peanut survival by reducing the relative abundance of *Fusarium* (Figure 2) and the abundance of plant pathogens (Figure 4b). This is consistent with the findings of previous studies on biochar inhibiting the abundance of soil pathogens and improving the yield and quality of ginseng [36,37].

Meanwhile, previous studies found that the bacteria that significantly decreased in bulk soil after continuous monocropping were mainly related to nitrogen cycling (i.e., *Rhizocola* and *Sorangium*) [38,39], while the significantly increased bacteria were mainly related to cellular degradation (*Arthrobacter*) [40], phosphorus cycling [41], and the inhibition of pathogenic fungi (*Fusarium*) [37].

A global meta-analysis indicated that biochar additions to soils overall increased the N- and P-cycling enzyme activities by 14% and 11%, respectively, while also reducing the C-cycling enzyme activities by 6.3% [42]. Compared to traditional compost, in line with the above studies, the results of biochar demonstrated a significant enhancement in soil extracellular enzyme activity (Table 4). Moreover, biochar–compost also substantially improved enzyme activity and extracellular enzyme activity diversity (H’ index). The findings revealed a significant increase in the functional diversity of enzymes in carbon-based organic fertilizers. Additionally, there was a noticeable rise in the ratio of C-cycle enzyme activity to N-cycle and P-cycle enzyme activity. This suggests that microorganisms utilize the carbon matrix to acquire energy for growth, reducing nutrient competition and indicating an enhanced nutrient availability due to the incorporation of biochar. Moreover, biochar contains approximately 70–90% stabilized carbon, and compost contains 2–14% stabilized carbon [43]. When biochar is applied to soil, some unstable carbon can become a carbon source for microbial growth and metabolism [44]. The labile C fraction of biochar may induce a priming effect to a rapid increase in the C-cycling SEAs during early or short-term experiments [45]. The high SOC should increase microbial and enzyme activities under biochar additions through the potential co-metabolism of biochar with SOC mineralization [46].

Overall, peanut production and quality were promoted through extracellular enzyme function and microbial community improvement in the continuously cropped peanut fields under biochar–compost treatment. In other words, biochar–compost addition could be a promising tool to enhance the continuous production of peanuts.

## 4. Materials and Methods

### 4.1. Experimental Site and Soil Condition

**Figure 5:**
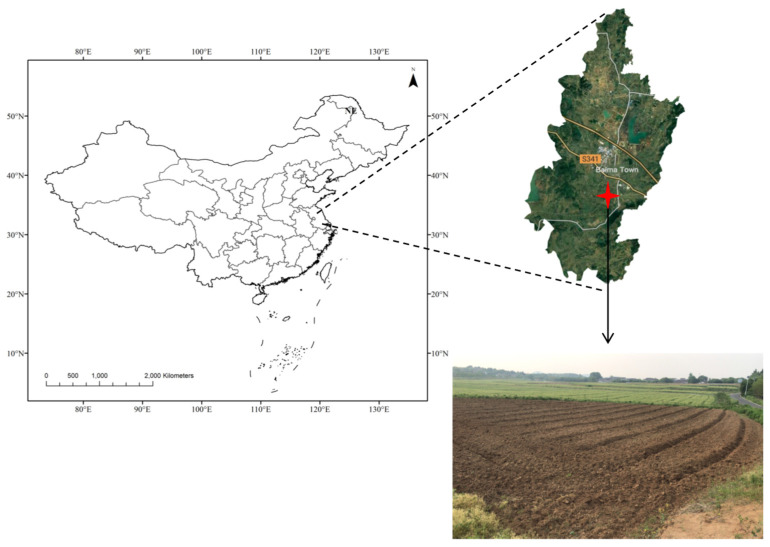
Site location of the experiment.

The experimental site was located in Fushan village (31°39′28″ N, 119°9′14″ E), Lishui District, Nanjing Municipality, Jiangsu Province, China (Figure 5). Under a subtropical monsoon climate, the local area has a mean annual temperature of 16.4 °C, an annual sunshine time of 1969 h, and frost-free days of 224 days, as well as precipitation of 1147 mm. Derived from the volcanic vents of ancient times, the soil was loamy clay classified as Alfisol in the US Soil Taxonomy (Soil Survey Staff, USDA 1994). The soil has been used for the continuous cropping of peanuts for 10 years.

The basic properties of the topsoil (0–20 cm) before the experiment were as follows: pH (H_2_O) of 6.03, bulk density of 1.25 g cm^−3^, soil organic carbon of 8.60 g kg^−1^, total N of 0.35 g kg^−1^, available P of 19.79 mg kg^−1^, and K of 91.93 mg kg^−1^, as well as a cation exchange capacity of 17.22 cmol kg^−1^.

### 4.2. Experimental Design

The field experiment was established in May 2022. The continuous cropping of peanuts was implemented as the current cropping system. The field trial was set in a randomized block design with five treatments, and each treatment was replicated three times. A treatment plot was laid out with an area of 20 m^2^ (6.7 m × 3.0 m), and a 0.2 m wide buffering strip was arranged between two adjacent treatment plots. The five treatments included the control with conventional organic manure (OM), woodchip biochar (WB), maize straw biochar (MB), a co-compost of woodchip biochar and pig manure (WBSC), and a co-compost of maize straw biochar and pig manure (MBSC). OM was composted using swine manure compensated with crop residue, purchased from Nanjing Ningliang Biofertilizer Technology Co. Ltd., Nanjing, China. WB and MB were produced via pyrolysis in a partially oxic vertical kiln in a temperature range of 450–550 °C, provided by Qinfeng Straw Technology Co., Ltd., Nanjing, China. WBSC and MBSC were co-composted at the Lishui Experimental Base of Nanjing Agricultural University. Briefly, swine manure, woodchip biochar/maize straw biochar, and zeolite were mixed at a mass ratio of 3:1:0.08 and allowed to compost for 8 weeks until maturity. These amendment materials were, respectively, air-dried and sieved through a 2 mm sieve, and homogenized before field amendment. The basic properties of the organic amendments are provided in Table S1.

One week before the sowing of peanut seeds, the amendment material was hand-spread onto the surface of the plot and evenly incorporated to a depth of approximately 15 cm with a wooden ranker following a tilling operation. All the farming performances were followed by local farmers and kept consistent throughout the treatment. As per conventional peanut production, all the plots were base-fertilized with a mineral compound fertilizer of N-P_2_O_5_-K_2_O (16-16-16, %) at 600 kg ha^−1^.

For the 2022 peanut season, presoaked peanut seeds (Xiaozi 3) were sown on 5 May 2022. The row space of plants in each plot was 35 cm, and the space between plants was 10 cm, resulting in a density of 180,000 holes per hectare. All the management practices were consistent across the treatment plots, following the farming practices for peanut production by the local farmers.

### 4.3. Plant Sampling and Analysis

Observation of plant traits was performed when the peanuts were harvested. In a plot, two rows were randomly selected to measure the pod and kernel yield. The roots were then washed with water and taken back to the laboratory for further analysis. Following our previous work [18], the contents of peanut kernel quality were determined with a near-infrared analyzer (DA7250, Perten, Hägersten, Sweden).

### 4.4. Soil Sampling and Analysis

While the peanuts were harvested in October 2022, peanut rhizosphere soil was collected for microbiome analysis, as per the protocols described by Butler et al. [47]. At each plot, ten peanut plant roots were randomly selected and gently hand-shaken to remove soil material attached, which were then collected and pooled as a peanut rhizosphere sample. For soil property analysis, a composite bulk topsoil (0–20 cm) sample was obtained with 5 individual sub-samples randomly collected using a stainless-steel tool in a treatment plot, following peanut harvest completion. All soil samples were sealed immediately in steel stainless cans, placed in an ice box, and shipped to the laboratory within 24 h. Upon arrival, the rhizosphere samples were immediately stored in the refrigerator at −80 °C before microbial DNA extraction.

In the laboratory, a fresh bulk soil sample was hand crashed, sieved to pass through a 2 mm sieve, and homogenized. Of this sample, one portion was air-dried and ground to pass, respectively, a 0.25 mm and a 0.15 mm sieve before physicochemical analyses, following the protocols described by Lu [48]. Another portion was stored at 4 °C for soil–water stable aggregate separation, as per Smith et al. [49], and for microbial biomass carbon and nitrogen measurement following the protocols described by Vance et al. [50], as well as for analysis of the soil extracellular enzyme activities (EEAs). In detail, EEAs of α-Glucosidase, β-Glucosidase, β-Xylosidase, cellobiohydrolase, N-acetyl-glucosaminidase, Polyphenol oxidase, and Peroxidase were performed, respectively, following the methods reported by Deforest [51] and German et al. [52].

### 4.5. DNA Extraction and Real-Time qPCR Analysis

A fresh sample (0.5 g) of rhizosphere stored at −80 °C was extracted to obtain total DNA with a Power Soil™ DNA Isolation Kit (Mo Bio Laboratories Inc., Carlsbad, CA, USA) (https://www.mobio.org/, accessed on 10 March 2025). This extraction was performed following the manufacturer’s instructions for the kit. Then, a qPCR was performed with 12.5 μL of SYBR premix EX Taq™ (Takara Shuzo, Otsu, Japan) in a total volume of 25 μL containing 10 ng DNA, 0.2 μM of a primer, and 0.2 mg mL^−1^ BSA. For bacteria and fungi, respectively, triplicate tenfold dilutions of plasmid DNA harboring cloned target genes were used to generate standard curves. Following each assay, a melting curve analysis was carried out to exclude amplifications from primer–dimers or other artifacts. In this study, the qPCR amplification efficiency was 103% for the bacterial 16S rRNA gene and 98% for the fungal ITS gene, respectively, with an R^2^ value > 0.99.

### 4.6. Illumina Hiseq Sequencing and Bioinformatics Analysis

Using the Illumina HiSeq platform, bacterial and fungal community compositions were further explored with sequencing target amplicons. Therein, the primer pair of 341F/806R was used to target the V3-V4 region of bacterial 16S rRNA genes, while that of ITS1F/ITS2R was used to target the fungal ITS region. The PCR products were then subject to gel electrophoresis in the presence of 2% (*w*/*v*) agarose. The obtained bands were purified with the AxyPrep DNA Gel Extraction Kit (Axygen Biosciences, CA, USA) (www.axygenbio.com, accessed on 10 March 2025), and their quantification was performed using the QuantiFluorTM-ST (Promega-GloMax Promega QuantiFluor, WI, USA). Hereby, the purified amplicons were concentrated on Illumina HiSeq in an isomolar concentration and sequenced as per the standard protocols detailed in the Supplementary Information.

Following Caporaso et al. [53] and Edgar [54], the obtained raw sequences were trimmed with the QIIME (quantitative insights into microbial ecology) software (QIIME Pipeline Version 1.8.0) and UPARSE pipeline. For this purpose, the sequences were screened for quality by eliminating barcodes, primers, and low-quality sequences. The remaining sequences were translated into amino acids using analysis from the Fun Gene Pipeline. Chimeric sequences and singletons were also removed using the UCHIME algorithm. The high-quality sequences were clustered into operational taxonomic units (OTUs) at a 97% similarity cut off. The BLAST algorithm was used to retrieve the NCBI GenBank database, and the representative sequences of an OTU were classified and identified. Data of the resultant OTUs were input into the QIIME software to calculate the rarefaction curves and the community diversity indices. Based on the OTU data, community functions were predicted using PICRUSt46 coupled with the KEGG Orthology classification scheme for bacteria and using the FUNGuild for fungi, respectively.

### 4.7. Statistical Analysis

All data were expressed as the means plus/minus standard deviation of a treatment plot, processed with Microsoft Excel (Version 2019). All statistical analyses were performed with ANOVA using SPSS software (Version 20.0). Duncan’s test was used to determine the significance of differences among the means, and Pearson’s test was used to assess the significance of a correlation. A difference among treatments is defined as significant at *p* < 0.05. Principal coordinate analysis (PCoA) was used to visualize the microbial community structure among the treatments based on a Bray–Curtis distance matrix.

## 5. Conclusions

In this study, peanut yield and quality were greatly improved through the enhanced activity and function of extracellular enzymes associated with C-, N-, and P-cycling, resulting in a manipulated network of rhizosphere microorganisms against pathogenic disease. While the microbial community structure shifted mainly to fungi, biochar and biochar–compost amendment led to a reduction in the pathotrophic fungi abundance. Furthermore, biochar–compost exerted better effects than pure biochar in terms of an increase in production, a promotion in soil aggregation, and an enhancement in soil nutrient cycling. Therefore, biochar-based amendment could be a strategic solution to regenerate the soil health and quality production of functional root crops while enhancing soil carbon storage in continuously cropped soils. This study is a short-term experiment, while long-term field studies of the biochar-based amendments on the improvement of soil biotic hazards need to be explored in the future.

## Figures and Tables

**Table 1:** Peanut yield and yield components at harvest under biochar and biochar–compost amendment.

Treatment	Pod Yield (kg ha^−1^)	Plant Height (cm)	Pod Number (plant^−1^)	Pod Weight (g plant^−1^)	Kernel Weight (g 100 kerner^−1^)	Pod Weight (g 100 pod^−1^)	Kernel to Pod Ratio (%)	Survival Rate (%)
OM	3039.03 ± 189.43 b	59.61 ± 3.82 b	44.33 ± 12.18 b	51.01 ± 10.98 c	68.20 ± 3.94 a	104.67 ± 4.73 b	65.13 ± 0.83 d	89.44 ± 2.68 b
WB	3715.24 ± 167.59 a	63.50 ± 2.26 a	54.00 ± 11.52 ab	53.50 ± 22.17 bc	78.71 ± 3.04 a	111.01 ± 6.24 ab	70.96 ± 1.99 ab	95.83 ± 1.67 a
MB	3469.64 ± 257.09 ab	63.21 ± 5.67 a	52.17 ± 8.30 ab	53.83 ± 15.28 bc	81.90 ± 8.40 a	120.67 ± 12.66 a	67.89 ± 0.38 c	95.28 ± 1.73 a
WBSC	3736.45 ± 380.94 a	64.82 ± 2.37 a	64.67 ± 12.79 a	71.40 ± 10.97 ab	79.22 ± 4.53 a	110.33 ± 5.03 ab	71.76 ± 1.15 a	95.83 ± 2.20 a
MBSC	3578.16 ± 346.83 a	63.88 ± 2.94 a	59.83 ± 7.08 a	68.25 ± 10.47 a	83.47 ± 6.05 a	119.67 ± 6.51 ab	69.70 ± 1.36 abc	95.56 ± 2.41 a

OM, amendment at 20 t ha^−1^ of conventional organic manure; WB and MB, amendment at 20 t ha^−1^ of woodchip biochar and maize straw biochar respectively; WBSC and MBSC, amendment at 20 t ha^−1^ of co-compost of woodchip biochar and pig manure, and co-compost of maize straw biochar and pig manure respectively. Different letters in a single column indicate significant differences among the treatments at *p* < 0.05. Data were presented as mean ± standard deviation (n = 3).

**Table 2:** Changes in peanut kernel quality (%) under the biochar and biochar–compost amendment.

Treatment	Fat	Protein	Oleic Acid	Linoleic Acid	Sugar	Cane Sugar	Soluble Sugar
OM	47.58 ± 0.92 a	27.36 ± 0.75 b	51.07 ± 1.52 b	29.73 ± 1.14 a	14.96 ± 0.79 ab	2.07 ± 0.11 ab	3.01 ± 0.35 c
WB	47.59 ± 0.64 a	28.71 ± 0.51 ab	51.72 ± 0.61 b	30.22 ± 1.05 a	15.15 ± 0.24 ab	2.30 ± 0.35 a	3.68 ± 0.11 b
MB	46.26 ± 0.87 a	30.44 ± 1.04 a	51.54 ± 1.20 b	30.44 ± 0.60 a	16.18 ± 0.32 a	2.51 ± 0.36 a	4.33 ± 0.12 a
WBSC	48.06 ± 1.77 a	29.00 ± 0.31 ab	53.77 ± 1.01 a	27.03 ± 1.54 b	13.98 ± 0.53 b	1.95 ± 0.08 b	3.16 ± 0.10 c
MBSC	48.45 ± 0.69 a	29.10 ± 0.83 ab	53.21 ± 1.82 a	27.58 ± 1.29 b	14.8 ± 0.26 b	1.96 ± 0.17 b	3.41 ± 0.22 ab

OM, amendment at 20 t ha^−1^ of conventional organic manure; WB and MB, amendment at 20 t ha^−1^ of woodchip biochar and maize straw biochar respectively; WBSC and MBSC, amendment at 20 t ha^−1^ of co-compost of woodchip biochar and pig manure, and co-compost of maize straw biochar and pig manure respectively. Different letters in a single column indicate significant differences among the treatments at *p* < 0.05. Data were presented as mean ± standard deviation (n = 3).

**Table 3:** Changes in soil properties under the biochar and biochar–compost amendment.

Treatment	pH (H_2_O)	BD	SOC	Total N	Available P	Available K	CEC	MBC	MBN
		(g cm^−3^)	(g kg^−1^)	(g kg^−1^)	(mg kg^−1^)	(mg kg^−1^)	(cmol kg^−1^)	(mg kg^−1^)	(mg kg^−1^)
OM	6.49 ± 0.50 bc	1.39 ± 0.14 a	9.70 ± 1.00 c	0.39 ± 0.03 b	26.53 ± 3.17 b	108.54 ± 7.69 b	22.70 ± 2.41 a	137.89 ± 11.25 b	21.21 ± 2.04 c
WB	7.37 ± 0.24 a	1.30 ± 0.11 a	16.26 ± 0.78 a	0.38 ± 0.04 b	28.71 ± 2.98 ab	131.26 ± 6.05 a	23.95 ± 0.97 a	132.77 ± 12.5 b	22.04 ± 3.92 bc
MB	7.34 ± 0.19 a	1.32 ± 0.15 a	16.32 ± 1.32 a	0.45 ± 0.04 ab	31.84 ± 2.74 a	148.39 ± 18.34 a	23.38 ± 3.14 a	162.80 ± 14.56 a	24.02 ± 2.05 bc
WBSC	6.82 ± 0.29 ab	1.29 ± 0.10 a	9.77 ± 0.86 c	0.47 ± 0.02 a	27.33 ± 2.44 ab	96.80 ± 9.47 b	24.28 ± 1.80 a	177.90 ± 3.04 a	27.29 ± 4.01 ab
MBSC	6.70 ± 0.30 b	1.22 ± 0.09 a	11.33 ± 1.22 bc	0.48 ± 0.04 a	26.27 ± 2.61 b	103.97 ± 7.14 b	23.15 ± 3.08 a	189.01 ± 14.96 a	24.89 ± 3.88 abc

OM, amendment at 20 t ha^−1^ of conventional organic manure; WB and MB, amendment at 20 t ha^−1^ of woodchip biochar and maize straw biochar respectively; WBSC and MBSC, amendment at 20 t ha^−1^ of co-compost of woodchip biochar and pig manure, and co-compost of maize straw biochar and pig manure respectively. Different letters in a single column indicate significant differences among the treatments at *p* < 0.05. Data were presented as mean ± standard deviation (n = 3).

**Table 4:** Activities of soil extracellular enzyme activity under the biochar and biochar–compost amendment.

Treatment	α-Glucosidase (nmol g^−1^ h^−1^)	β-Glucosidase (nmol g^−1^ h^−1^)	β-Xylosidase (nmol g^−1^ h^−1^)	β-Cellobiohydrolase (nmol g^−1^ h^−1^)	N-Acetyl-Glucosaminidase (nmol g^−1^ h^−1^)	Acid Phosphatase (nmol g^−1^ h^−1^)	Sulfatase (nmol g^−1^ h^−1^)
OM	11.95 ± 0.64 d	104.86 ± 4.69 d	11.93 ± 6.36 c	22.54 ± 0.96 b	52.69 ± 2.90 c	449.15 ± 23.69 c	4.98 ± 0.26 a
WB	14.21 ± 0.77 c	152.68 ± 8.49 b	17.27 ± 0.84 b	31.99 ± 1.57 a	56.45 ± 2.85 b	518.87 ± 34.26 ab	6.58 ± 0.35 a
MB	17.18 ± 0.45 b	144.08 ± 4.59 c	17.39 ± 0.58 b	22.68 ± 1.06 b	58.02 ± 2.08 b	513.83 ± 16.42 b	5.19 ± 0.17 a
WBSC	17.18 ± 0.45 b	152.59 ± 4.59 b	21.51 ± 0.34 a	23.88 ± 0.37 b	63.61 ± 1.84 a	515.46 ± 15.40 b	5.39 ± 2.70 a
MBSC	25.78 ± 0.62 a	167.87 ± 3.52 a	22.39 ± 0.47 a	31.89 ± 1.56 a	62.20 ± 1.23 a	553.87 ± 10.47 a	6.68 ± 0.13 a
**Treatment**	**Polyphenol Oxidase** **(μmol g^−1^ h^−1^)**	**Peroxidase** **(μmol g^−1^ h^−1^)**	**H’ Index**	**Hydrolase/Non-Hydrolase**	**C/N Cycling**	**C/P Cycling**	**N/P Cycling**
OM	1.33 ± 0.74 a	4.22 ± 0.89 c	1.09 ± 0.03 c	0.44 ± 0.02 d	2.87 ± 0.08 c	0.34 ± 0.01 d	0.12 ± 0.00 a
WB	1.81 ± 0.41 a	5.65 ± 0.28 b	1.15 ± 0.02 b	0.51 ± 0.02 b	3.84 ± 0.28 a	0.42 ± 0.01 b	0.11 ± 0.01 a
MB	1.98 ± 0.34 a	5.38 ± 0.48 b	1.13 ± 0.01 b	0.49 ± 0.00 c	3.47 ± 0.03 b	0.39 ± 0.00 c	0.11 ± 0.00 a
WBSC	1.83 ± 0.35 a	5.28 ± 0.51 b	1.16 ± 0.02 b	0.53 ± 0.00 b	3.37 ± 0.01 b	0.42 ± 0.01 b	0.12 ± 0.00 a
MBSC	1.98 ± 0.21 a	7.01 ± 0.74 a	1.20 ± 0.01 a	0.55 ± 0.00 a	3.99 ± 0.11 a	0.45 ± 0.00 a	0.11 ± 0.00 a

OM, amendment at 20 t ha^−1^ of conventional organic manure; WB and MB, amendment at 20 t ha^−1^ of woodchip biochar and maize straw biochar respectively; WBSC and MBSC, amendment at 20 t ha^−1^ of co-compost of woodchip biochar and pig manure, and co-compost of maize straw biochar and pig manure respectively. Different letters in a single column indicate significant differences among the treatments at *p* < 0.05. Data were presented as mean ± standard deviation (n = 3).

## Data Availability

Data are contained within the article or Supplementary Materials.

## Supplementary Materials

The following supporting information can be downloaded at: https://www.mdpi.com/article/10.3390/plants14060922/s1, Table S1 Basic properties of the amendment materials used in this study; Table S2 Changes in soil micro-element under the biochar and biochar–compost amendment. Different letters in a single column indicate significant differences among the treatments at p < 0.05. Data were presented as mean ± standard deviation (n = 3).
