# Supplementary material for: Biochar Amendment Increases Peanut Production Through Improvement of the Extracellular Enzyme Activities and Microbial Community Composition in Replanted Field
Source: Plants (Basel). 2025 Mar 15;14(6):922. doi: 10.3390/plants14060922 (PMC11945357; doi:10.3390/plants14060922)
Supplement: Supplementary file 1 [file plants-14-00922-s001.zip › plants-3504733-supplementary.pdf]

Table S1 Basic properties of the amendment materials used in this study

Material	pH (H_2O)	Org. C (g kg^{-1})	Total N (g kg^{-1})	Total P (g kg^{-1})	Total K (g kg^{-1})
OM	7.73	302.30	9.30	13.40	15.60
WB	10.15	531.92	2.97	3.08	7.22
MB	9.95	463.63	6.78	5.55	25.71
WBSC	8.37	178.34	10.43	10.34	11.27
MBSC	8.06	154.73	12.18	11.09	11.95

OM, organic fertilizer; WB, woodchip biochar; MB, maize straw biochar; WBSC, co-compost of woodchip biochar and pig manure; MBSC, co-compost of maize straw biochar and pig manure

Table S2 Changes in soil micro-element under the biochar and biochar-compost amendment

	Al ($g\ kg^{-1}$)	Cu ($mg\ kg^{-1}$)	Fe ($g\ kg^{-1}$)	Mn ($g\ kg^{-1}$)	Zn ($mg\ kg^{-1}$)	Mo ($mg\ kg^{-1}$)	S ($g\ kg^{-1}$)
OM	31.23±0.57a	19.14±1.09a	25.38±1.67b	0.51±0.09a	45.64±3.32b	1.95±0.19b	3.38±0.24b
MB	28.83±1.52a	19.63±1.11a	32.17±0.62a	0.61±0.14a	49.15±1.99ab	2.03±0.17ab	4.02±0.32a
WB	29.33±1.15a	21.50±0.52a	32.01±0.94a	0.60±0.14a	48.65±2.08ab	2.03±0.23ab	4.17±0.52a
MBSC	30.46±1.44a	21.95±0.26a	30.85±1.22a	0.57±0.15a	52.86±2.82a	2.35±0.20a	3.56±0.44ab
WBSC	31.65±0.82a	22.98±2.84a	29.74±1.21a	0.59±0.08a	50.20±2.43a	2.24±0.16a	3.60±0.31ab

OM, organic fertilizer; WB, woodchip biochar; MB, maize straw biochar; WBSC, co-compost of woodchip biochar and pig manure; MBSC, co-compost of maize straw biochar and pig manure. Different letters in a single column indicate significant differences among the treatments at $p < 0.05$. Data were presented as mean ± standard deviation (n = 3).
